# Proteomic profiling of L-cysteine induced selenite resistance in *Enterobacter *sp. YSU

**DOI:** 10.1186/1477-5956-7-30

**Published:** 2009-08-28

**Authors:** Ashley Jasenec, Nathaniel Barasa, Samatha Kulkarni, Nabeel Shaik, Swarnalatha Moparthi, Venkataramana Konda, Jonathan Caguiat

**Affiliations:** 1 Department of Biological Sciences, Proteomics/Genomics Research Group, Youngstown State University, One University Plaza, Youngstown, OH 44555, USA

## Abstract

**Background:**

*Enterobacter *sp. YSU is resistant to several different heavy metal salts, including selenite. A previous study using M-9 minimal medium showed that when the selenite concentration was 100,000 times higher than the sulfate concentration, selenite entered *Escherichia coli *cells using two pathways: a specific and a non-specific pathway. In the specific pathway, selenite entered the cells through a yet to be characterized channel dedicated for selenite. In the non-specific pathway, selenite entered the cells through a sulfate permease channel. Addition of L-cystine, an L-cysteine dimer, appeared to indirectly decrease selenite import into the cell through the non-specific pathway. However, it did not affect the level of selenite transport into the cell through the specific pathway.

**Results:**

Growth curves using M-9 minimal medium containing 40 mM selenite and 1 mM sulfate showed that *Enterobacter *sp. YSU grew when L-cysteine was present but died when it was absent. Differential protein expression analysis by two dimensional gel electrophoresis showed that CysK was present in cultures containing selenite and lacking L-cysteine but absent in cultures containing both selenite and L-cysteine. Additional RT-PCR studies demonstrated that transcripts for the sulfate permease genes, *cysA*, *cysT *and *cysW*, were down-regulated in the presence of L-cysteine.

**Conclusion:**

L-cysteine appeared to confer selenite resistance upon *Enterobacter sp. *YSU by decreasing the level of selenite transport into the cell through the non-specific pathway.

## Introduction

Selenium is an important cofactor in some mammalian and bacterial enzymes [1-5]. It is found in mammalian glutathione peroxidase [6] and bacterial formate dehydrogenase [7] in the form of selenocysteine. A series of *Escherichia coli *(*E. coli*) proteins, SelA, SelB, SelC and SelD, incorporate selenium into selenocysteine which is then inserted into proteins. The mechanisms that transport selenite into the cell are not well understood. Once it enters the cell, it may be reduced to selenide by glutathione [8] or thioredoxin [9]. Then, SelD, a selenophosphate synthetase, catalyzes a reaction with selenide and ATP to make selenophosphate [10,11]. SelA, a selenocysteine synthetase, uses selenophosphate to convert serine to selenocysteine [12], which is carried by SelC, a special tRNA [13]. Finally, SelB, a translation factor, inserts selenocysteine at a UGA stop codon. The mRNAs of proteins that encode selenocysteine also contain a special stem loop structure or selenocysteine insertion sequence (SECIS) which stalls the ribosome and allows SelB to insert selenocysteine at the UGA stop codon [14,15].

Although selenium is an important cofactor, too much can be toxic. Selenium inhibits the growth of *E. coli *when it is present at overabundant concentrations as selenite, but non-toxic when it is present as elemental selenium [16]. Selenite reacts with glutathione and other thiol containing proteins to produce highly reactive superoxides [16-19], which may kill the cells by damaging their DNA and lipids [18,20].

Selenite and selenate resistant bacteria appear to remove excess selenium by reducing it to elemental selenium or methylating it [5]. A strain of *Stenotrophomonas maltophilia *(*S. maltophilia*) isolated from a selenium-contaminated drainage pond reduces selenate and selenite to elemental selenium and deposits it near the cell surface and in the surrounding growth medium [21]. In addition, *Cupriavidus metallidurans *(*C. metallidurans*) CH34, a multi-metal resistant strain [22] from a zinc contaminated site, is resistant to selenate and selenite and reduces both oxyanions to elemental selenium and alkyl selenide under aerobic conditions [23,24]. A *C. metallidurans *CH34 protein, DedA, which was identified by transposon mutagenesis, may be involved in selenite uptake [25]. A set of *E. coli *proteins, YgfK, YgfM and YgfN, which were also identified by transposon mutagenesis, act as an enzyme that reduces selenate to elemental selenium [26]. Finally, some bacterial strains also methylate selenite and selenate by encoding a thiopurine methyltransferase, which produces volatile dimethyl selenide and dimethyl diselenide [27,28].

Understanding how selenite enters the cell is important to understanding the mechanisms of selenite resistance. An early study in *E. coli *shows that selenite and sulfate follow a Michaelis-Menten model when they are transported into the cell and compete competitively with each other [29]. Using a low sulfate, M-9 minimal medium containing 10 μM sulfate and ^75^Se-labeled selenite mixed with 1 M unlabeled selenite, Muller *et al *define two pathways for selenium incorporation in *E. coli*: (1) specific and (2) non-specific [30,31]. In the specific pathway, selenite enters the cell through a pathway dedicated for selenite and becomes incorporated into selenocysteine intentionally. In the non-specific pathway, selenite enters the cell through the sulfate transport system and becomes incorporated unintentionally to produce selenocysteine instead of L-cysteine. When 10 μg/ml of L-cystine, an L-cysteine dimer, is added to the medium, the wild type *E. coli *strain incorporates selenium randomly into cellular proteins, presumably via the non-specific pathway. However, when 60 μg/ml L-cystine is added to the medium, the amount of randomly incorporated selenium in wild type *E. coli *decreases dramatically. These results suggest that feedback inhibition blocks both the biosynthesis pathway for L-cysteine and the accidental incorporation of selenium into proteins. In support of this hypothesis, *cysK *mutants, which lack the ability to add sulfide to O-acetylserine to generate L-cysteine, fail to randomly incorporate radioactive selenite into protein through the non-specific pathway [30,31]. Thus, under most conditions when selenite concentrations are lower than sulfate concentrations, selenite is excluded from the non-specific pathway. However, when selenite concentrations are higher than sulfate concentrations, selenite is transported into the cell through the non-specific pathway instead of sulfate.

*Enterobacter sp. *YSU is a multi-metal resistant strain that grows in the presence of mercury, cadmium, zinc, gold, silver, arsenic and selenium [32]. It does not grow in M-9 minimal medium containing 1 mM sulfate and 40 mM selenite. However, growth inhibition is relieved by the addition of L-cysteine. Here, we define L-cysteine-dependent selenite resistance in *Enterobacter sp. *YSU using growth curves, 2-D gel electrophoresis and reverse-transcriptase-polymerase chain reactions (RT-PCR).

## Results

### Requirement of L-cysteine for selenite resistance

**Figure 1:**
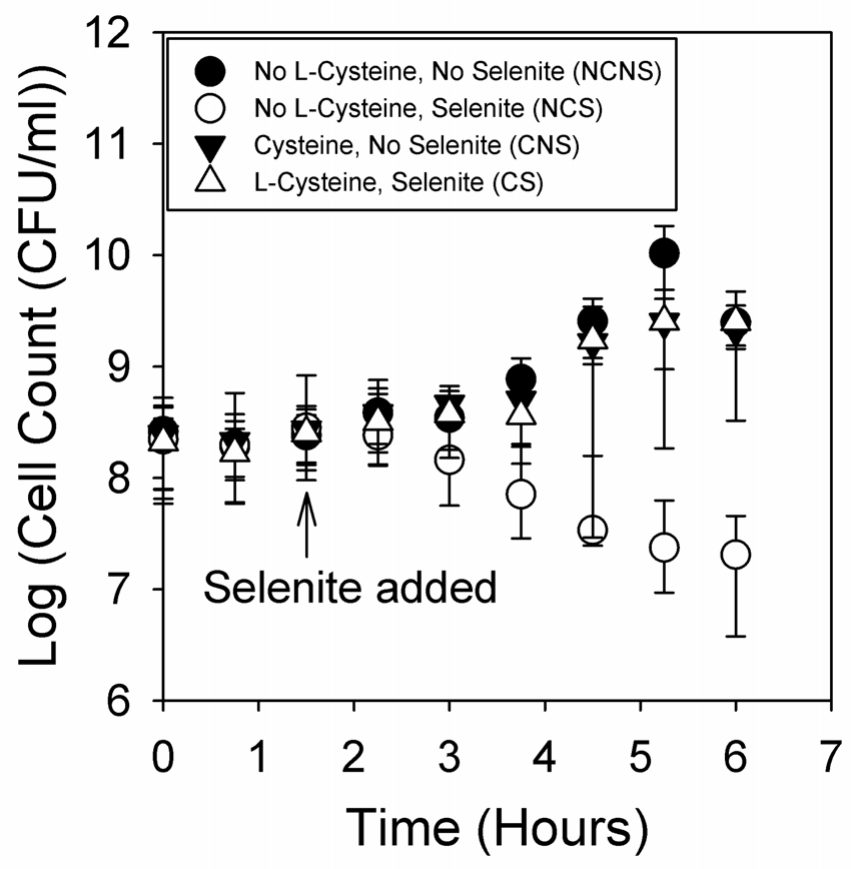
**Viable cell count growth curves**. Overnight cultures were diluted 1:20 in fresh M-9 minimal medium and grown at 37°C. After 1.5 hours of growth, selenite or water was added to each culture. Samples were diluted and spread on plates containing LB medium every 45 minutes. Values at each time point are the average of at least 3 different experiments, and error was calculated using the student t test at a 95% confidence level.

*Enterobacter sp. *YSU was grown in four M-9 minimal medium cultures. During early log phase after 1.5 hours of growth, selenite or an equal volume of water was added to give the following growth conditions: no L-cysteine and no selenite (NCNS), no L-cysteine and selenite (NCS), L-cysteine and no selenite (CNS), and L-cysteine and selenite (CS). Every 45 minutes, culture samples were diluted and plated to follow viable cell count versus time (Fig 1). Each experiment was performed four times, and each plotted point was the average of cell counts from at least three of the four experiments. Both conditions without selenite (NCNS and CNS) demonstrated normal growth curves. The bacteria in the culture lacking L-cysteine and containing selenite (NCS) were killed by the selenite, and 3.5 hours after adding selenite, the number of viable cells decreased on average by 98%. The culture containing both L-cysteine and selenite (CS), on the other hand, demonstrated a normal growth curve, and 3.5 hours after adding selenite, the number of viable cells increased on average by 890%. These results clearly showed that without L-cysteine, 40 mM selenite is toxic to *Enterobacter sp. *YSU.

### Growth curves used for proteomic analysis

Two stationary phase cultures of *Enterobacter sp. *YSU grown in M-9 minimal medium containing and lacking 40 μg/ml L-cysteine were diluted 1:20 into fresh medium containing and lacking L-cysteine. Growth was followed by measuring turbidity every 30 minutes. After 2.5 hours of growth, the NCNS and CNS samples were taken for protein analysis. The cultures were split into equal volumes, and selenite or water was added to give the four conditions. One hour after selenite was added, the NCS and CS samples were taken for protein analysis.

**Figure 2:**
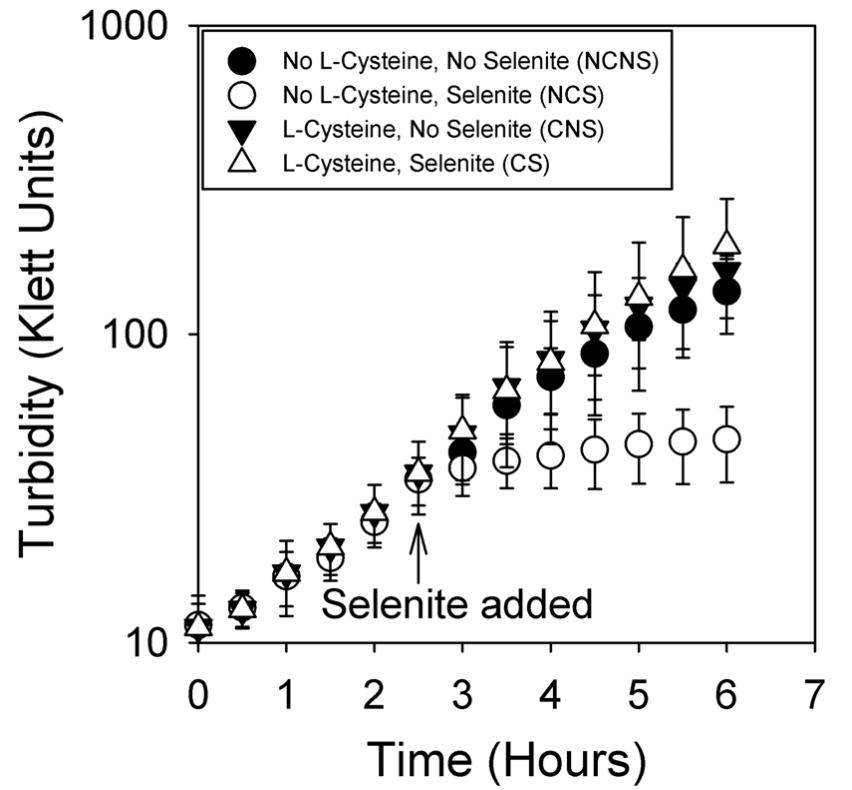
**Turbidity growth curves**. Overnight cultures were diluted 1:20 in fresh M-9 minimal medium and grown at 37°C. After 2.5 hours of growth, selenite or water was added to each culture. Turbidity was measured every 30 minutes using a Klett colorimeter. Values at each time point are the average of 6 different experiments, and error was calculated using the student t test at a 95% confidence level.

These growth curves also showed that L-cysteine played a role in selenite resistance (Fig 2). The two positive control cultures, NCNS and CNS, demonstrated a typical growth curve and increased on average to final cell densities of 160 ± 19 and 138 ± 38 Klett units, respectively. The cultures that lacked L-cysteine and contained selenite, NCS, increased in cell density on average by only12 Klett units to 46 ± 13 Klett units 3.5 hours after selenite was added. However, the cultures that contained L-cysteine and selenite (CS) were able to grow. This culture increased in cell density to 193 ± 81 Klett units 3.5 hours after selenite was added.

### Two-dimensional gel electrophoresis (2DGE)

**Figure 3:**
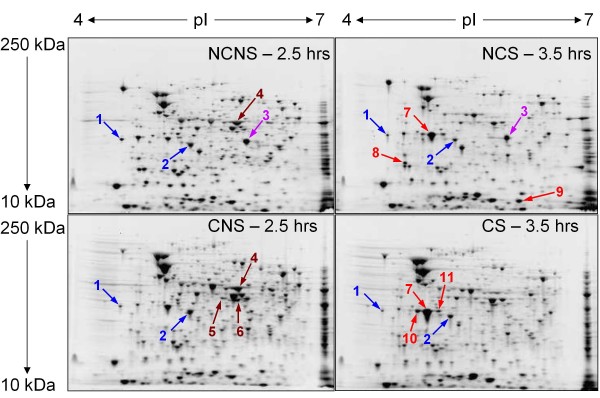
**Negative images of *Enterobacter *sp. YSU total protein separated by 2DGE over a pI range of 4-7**. Cultures were grown as in Fig 2. Cultures grown with No L-Cysteine and No Selenite (NCNS) and with L-Cysteine and No Selenite (CNS) were harvested after 2.5 hours of growth. The cultures grown with No L-Cysteine and Selenite (NCS) and with L-Cysteine and Selenite (CS) were harvested after 3.5 hours of growth. Spots identified with blue arrows appeared with equal intensities under all 4 conditions. The spot identified by a purple arrow appeared at a higher intensity in gels containing samples from cells grown in the absence of L-cysteine. Spots identified by dark red arrows appeared at higher intensities in gels containing samples from cells grown in the absence of selenite. Spots identified by red arrows appeared at higher intensities in gels containing samples from cells grown in the presence of selenite. The spot numbers correspond to the spot numbers in Table 1.

Protein samples from all 4 culture conditions were analyzed by 2DGE between the pI ranges of 4 and 7 (Fig 3). Differences in protein expression were detected by comparing the size and intensity of the same spot on each gel. Spots that appeared with equal intensities or higher intensities compared to other spots in the same location on other gels were excised, digested with trypsin and analyzed by mass spectrometry (Nano-LC/MS/MS analysis). Analysis of the peptides from each spot using the Mascot software package [33] identified significant matches to known proteins. For a match to be considered significant, it contained at least two different peptide fragments matching part of an entire known sequence.

### Proteins in spots that appeared at equivalent intensities in gels of samples prepared from cells grown under all four conditions

Two landmark spots, which are identified by blue arrows (Fig 3), appeared at equal intensities in all four gels and were selected to demonstrate accuracy. Spot 1 from all four gels contained peptides that matched to an OmpF porin from *Enterobacter cloacae *with Mascot scores ranging from 496-520 and sequence coverages ranging from 22-23% (Table 1). Spot 2 from all four gels contained peptides that matched to a protein chain elongation factor EF-Ts from *Salmonella typhimurium *(*S. typhimurium*) with Mascot scores ranging from 654-1090 and sequence coverages ranging from 44-60%.

**Table 1:** Identification of select protein spots that appeared under all four conditions (NCNS, NCS, CNS and CS)^a^.

**Spot #/Condition**	**Protein Name**	**NCBI Accession (Version)**	**Theo. Mr/pI (kDa)**	**Estimated Expt. Mr/pI (kDa)**	**Species**	**Mascot Score**	**NP/PD**	**MS/MS Peptide Sequence**	**SC (%)**
1 Equally intense under all conditions	OmpF porin	CAC48383 gi|15131544	4.63/38.4	4.6/30	*Enterobacter cloacae*	520	8/31	DGNKLDLYGK LDLYGK LAFAGLK FGDAGSFDYGR TGGLATYR AEQWATGLK YDANNIYLAALYGEMR NMSTYVDYQINQLKDDNK	22%
									
2 Equally intense under all conditions	protein chain elongation factor EF-Ts	AAL19181 gi|16418721	5.13/30.4	5.3/25	*Salmonella typhimurium*	1090	13/69	AEITASLVKELRER ALTEANGDIELAIENMRK KAGNVAADGVIKTK DAGFQAFADK VLDAAVAGK ITDVEVLK AQFEEER IGENINIR GADEELVK EYQVQLDIAMQSGKPK EHNADVTGFIRFEVGEGIEKVETDFAAEVAAMSK FEVGEGIEKVETDFAAEVAAMSK VETDFAAEVAAMSK	51%
									
3 NCNS & NCS	conserved hypothetical lipobinding protein	NP_758751 gi|27228700	5.57/29.5	6/32	*Erwinia pyrifoliae*	153	2/9	ISDIVENPK YKGAVIPVNN	7%
									
Only in NCS	branched-chain amino-acid aminotransferase	AAM83928 gi|21957021	6.22/36.9	6/32	*Yersinia pestis*	277	5/6	IYRMPVSQSVDELMEACR VAPNTIPTAAK AGGNYLSSLLVGSEAR DGILFTPPFTSSALPGITR ESLYLADEVFMSGTAAEITPVR	25%
									
Only in NCS	outer membrane protein II	AAA24807 gi|148368	4.88/25.7	6/32	*Enterobacter aerogenes*	104	2/3	LGYPVTDDLDVYTR SDVLFNFNK	9%
									
4 NCNS & NS	translation elongation factor EF-Tu	AAL22974 gi|16422703	5.24/43.5	5.8/35	*Salmonella typhimurium*	1143	18/64	TTLTAAITTVLAK AFDQIDNAPEEK GITINTSHVEYDTPTR NMITGAAQMDGAILVVAATDGPMPQTR EHILLGR QVGVPYIIVFLNK CDMVDDEELLELVEMEVR ELLSQYDFPGDDTPIVR AIDKPFLLPIEDVFSISGR VGEEVEIVGIK STCTGVEMFR KLLDEGR AGENVGVLLR FESEVYILSK GYRPQFYFR TTDVTGTIELPEGVEMVMPGDNIK MVVTLIHPIAMDDGLR TVGAGVVAK	64%
									
5, 6 NCNS & NS	translation elongation factor EF-Tu	AAL22974 gi|16422703	5.3/43.4	5.8/36	*Salmonella typhimurium*	666	10/19	TTLTAAITTVLAK AFDQIDNAPEEK NMITGAAQMDGAILVVAATDGPMPQTR ELLSQYDFPGDDTPIVR VGEEVEIVGIK AGENVGVLLR FESEVYILSK GYRPQFYFR MVVTLIHPIAMDDGLR TVGAGVVAK	30%
									
In 6 but not in 5	5-methyltetrahydropteroyltriglutamate-homocysteine methyltransferase	CAG76025 gi|49612575	5.69/38.9	5.8/36	*Erwinia carotovora*	173	4/6	ILNQEAR LAWEFAK ALQFVDADK AGIDIVSDGEQTR	10%
									
7, 10, 11 NCS & CS	outer membrane protein II	AAA24807 gi|148368	4.88/25.6	5.1/35	*Enterobacter aerogenes*	921	11/64	LGYPVTDDLDVYTRLGGMVWR ADTSNSIAGDDHDTGVSPVFAGGVEWAMTR LEYQWVNNIGDGATVGVRPDNGMLSVGVSYR FGQQEDAPVVAPAPAPAPEVQTK SDVLFNFNK ATLKPEGQQALDQLYTQLSNLDPK DGSVVVLGFTDR IGSDAYNQGLSEKR RAQSVVDYLVSK GMGESNPVTGSTCDNVKPR AALIDCLAPDR	86%
									
NCS & CS	OmpA	AAY18798 gi|62901665	5.19/37.1	5.1/35	*Enterobacter sakazakii*	700	10/54	DNTWYAGGK AQGVQLTAK LGYPVTDDLDVYTRLGGMVWR LGGMVWR ADSSSNIAGDDHDTGVSPVFAGGVEWAMTRDIAT SDVLFNFNK DGSVVVLGFTDR IGSDAYNQGLSEKR AQSVVDYLISK GMGESNPVTGNTCDNVKAR	40%
									
NCS & CS	putative membrane component hydrogenase	AAL20003 gi|16419585	5.6/37.6	5.1/35	*Salmonella typhimurium*	561	8/45	AQGVQLTAK LGYPITDDLDVYTR LGGMVWR SDVLFNFNK DGSVVVLGFTDR IGSDAYNQGLSEKR AQSVVDYLISK AALIDCLAPDRR	25%
									
Only in spot 7, NCS	cysK protein	AAA23654 gi|145686	5.64/34.6	5.1/35	*Escherichia coli*	245	4/9	LTLTMPETMSIER ALGANLVLTEGAK VIGITNEEAISTAR NIVVILPSSGER	16%
									
Only in spot 10	60 kDa chaperonin (groEL)	O66200 gi|6225120	4.85/56.5	5/35	*Pantoea agglomerans*	333	4/6	ANDAAGDGTTTATVLAQAIITEGLK AIAQVGTISANSDETVGK AMLQDIATLTGGTVISEEIGMELEK LAGLTAQNEDQNVGIK	15%
									
8 Only in NCS	putative tellurium resistance protein C	AAF36435 gi|7108483	4.56/20.5	4.8/22	*Escherichia coli*	516	6/14	AAPSMKNVLVGLGWDAR GDSDFIFYNNLTSSDGSVTHTGDNR TGEGDGDDESLKIK RQSFGQVSGAFIR LVNDDNQTEVAR YDLTEDASTETAMLFGELYR	52%
									
	similar to GroES protein	BAA25224 gi|2980925	4.90/9.3	4.8/22	*Klebsiella pneumoniae*	184	3/5	SAGGIVLTGSAAAK ILENGTVQPLDVK VGDIVIFNDGYGVK	46%
									
	outer membrane protein II	AAA24807 gi|148368	4.88/25.6	4.8/22	*Enterobacter aerogenes*	191	2/6	LGYPVTDDLDVYTRLGGMVWR FGQQEDAPVVAPAPAPAPEVQTK	18%
									
	OmpA	AAY18798 gi|62901665	5.19/37.1	4.8/22	*Enterobacter sakazakii*	165	2/4	AQGVQLTAK LGYPVTDDLDVYTRLGGMVWR	8%
									
9 Only in NCS	small heat shock protein	AAL22668 gi|16422381	5.23/15.7	6.5/10	*Salmonella typhimurium*	299	4/26	MRNFDLSPLYR SAIGFDR ERTYLYQGIAER GANLVNGLLYIELER	32%

^a ^Spot number corresponds to labeled spots in Fig 3; Condition refers to the gel(s) in which the spot or protein appeared at the greatest intensity; Protein name, matched protein description; NCBI Accession (Version), accession number and submission version of matched protein from NCBI database; Theo. pI/Mr (kDa), theoretical isoelectric point and molecular mass based on amino acid sequence of the identified protein; Expt. pI/Mr (kDa), experimental isoelectric point and molecular mass estimated from the 2DGE gels; Species, the bacterial species of the matched protein; Mascot score, score obtained from the Mascot search for each match; NP, the number of matched peptides; PD, the number of peptides detected; MS/MS Peptide Sequence, amino acid sequences of peptides identified by Nano-LC/MS/MS; SC, percent amino acid sequence coverage for the identified protein.

### Proteins in a spot that appeared at a higher intensity in gels of samples prepared from cells grown in the absence of L-cysteine

Spot 3, which is identified by a purple arrow (Fig 3), appeared at a higher intensity in the gels of samples prepared from cells grown in the absence of L-cysteine (NCNS and NCS). This spot contained peptides that matched to a conserved hypothetical lipobinding protein from *Erwinia pyrifoliae *with a Mascot score of 153 and a sequence coverage of 7% (Table 1). Spot 3 from the NCS gel also contained peptides that matched to two other proteins: a branched-chain amino-acid aminotransferase from *Yersinia pestis *with a Mascot score of 277 and a sequence coverage of 25% and an outer membrane protein II from *Enterobacter aerogenes *(*E. aerogenes*) with a Mascot score of 104 and a sequence coverage of 9%.

### Proteins in spots that appeared at higher intensities in gels of samples from cells grown in the absence of selenite

Three spots, which are identified by dark red arrows (Fig 3), appeared at higher intensities on gels that contained samples prepared from cells grown in the absence of selenite (NCNS and CNS). Spot 4 from the NCNS and CNS gels was unique. This spot contained peptides that matched to an EF-Tu protein from *S. typhimurium *with Mascot scores of 1143 and 1467 and sequence coverages of 64% and 52%, respectively (Table 1). In addition, spots 5 and 6 were more intense in gels containing samples prepared from cells grown in the absence of selenite (NCNS and CNS). They contained peptides that matched to EF-Tu from *S. typhimurium *with Mascot scores of 611 and 666 and sequence coverages of 25% and 30%, respectively. Spot 6 also contained peptides that matched to 5-methyltetrahydropteroyltriglutamate-homocysteine methyltransferase (MetE) from *Erwinia carotovora *with a Mascot score of 173 and a sequence coverage of 10%.

### Proteins in spots that appeared at higher intensities in gels of samples prepared from cells grown in the presence of selenite

Five spots, which are identified by red arrows (Fig 3), appeared at higher intensities in gels of samples prepared from cells grown in the presence of selenite (NCS and CS). Spot 7 was the largest in the CS gels. In the no L-cysteine, selenite gel (NCS), it contained peptides that matched to 4 proteins (Table 1): an outer membrane protein II from *E. aerogenes *with a Mascot score of 921 and a sequence coverage of 86%, OmpA from *E. sakazakii *with a Mascot score of 700 and a sequence coverage of 40%, a putative membrane component hydrogenase from *S. typhimurium *with a Mascot score of 561 and a sequence coverage of 25% and a CysK protein from *E. coli *with a Mascot score of 245 and a sequence coverage of 16%. In the CS gels, it contained a similar set of peptides for 3 proteins: an outer membrane protein II from *E. aerogenes *with a Mascot score of 960 and a sequence coverage of 73%, OmpA from *E. sakazakii *with a Mascot score of 696 and a sequence coverage of 38% and a putative membrane component hydrogenase from *S. typhimurium *with a Mascot score of 549 and a sequence coverage of 25%. It lacked peptides that matched to CysK.

Under selenite sensitive condition (NCS), spots 8 and 9 were unique (Fig 3). They did not appear in gels of samples prepared from cells grown under the other three conditions. Spot 8 contained peptides that matched to 4 proteins (Table 1): a putative tellurium resistance protein C from *E. coli *[34] with a Mascot score of 516 and a sequence coverage of 52%, a protein similar to GroES from *Klebsiella pneumoniae *with a Mascot score of 184 and a sequence coverage of 46%, an outer membrane protein II from *E. aerogenes *with a Mascot score of 191 and a sequence coverage of 18% and OmpA from *E. sakazakii *with a Mascot score of 165 and a sequence coverage of 8%. Spot 9 contained peptides that matched to a small heat shock protein from *S. typhimurium *with a Mascot score of 299 and a sequence coverage of 32%.

Spots 10 and 11 were unique on the gels containing protein samples prepared from cells treated with selenite (NCS and CS). Spot 10 contained peptides that matched to 4 proteins: an outer membrane protein II from *E. aerogenes *with a Mascot score of 917 and a sequence coverage of 73%, OmpA from *E. sakazakii *with a Mascot score of 633 and a sequence coverage of 35%, a putative membrane component hydrogenase from *S. typhimurium *with a Mascot score of 501 and a sequence coverage of 22% and GroEL from *Pantoea agglomerans *with a Mascot score of 333 and a sequence coverage of 15%. Spot 11 contained peptides that matched to 3 proteins: outer membrane protein II from *E. aerogenes *with a Mascot score of 641 and a sequence coverage of 52%, OmpA from *E. sakazakii *with a Mascot score of 434 and a sequence coverage of 37% and a putative membrane component hydrogenase from *S. typhimurium *with a Mascot score of 362 and a sequence coverage of 20%. It lacked peptides for GroEL.

### Detection of cysK, cysE and sulfate permease transcripts in cells grown in the presence and absence of L-cysteine

**Figure 4:**
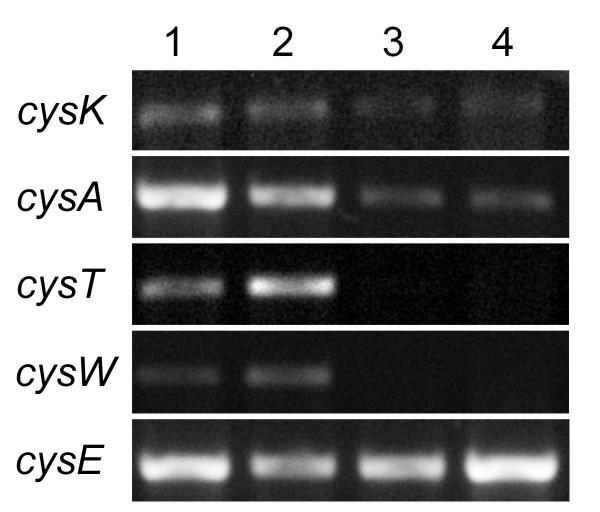
**Detection of the *cysK*, *cysA*, *cysT*, *cysW *and *cysE *transcripts using RT-PCR**. Cells were grown and harvested as in Fig 3. Equal volumes of cDNA synthesized from 0.5 μg of total RNA were used in PCR reactions containing primers specific for each gene, and 10 μl of each PCR reaction were analyzed by agarose gel electrophoresis. Lanes: (1) No L-Cysteine, No Selenite (NCNS); (2) No L-Cysteine, Selenite (NCS); (3) L-Cysteine, No Selenite (CNS); (4) L-Cysteine, Selenite (CS). The gene, *cysE*, was used as an internal control.

The absence of CysK in protein samples of cells grown in the presence of L-cysteine and selenite (CS) suggested that the addition of L-cysteine down-regulated the expression of the L-cysteine synthesis and the sulfate permease genes. To verify this hypothesis, *Enterobacter *sp. YSU was grown in M-9 minimal medium in the presence and absence of L-cysteine. The untreated samples (NCNS and CNS) were harvested immediately before selenite was added, and the selenite treated samples (NCS and CS) were harvested one hour after selenite was added. Equal amounts of purified RNA were converted to cDNA. Then, PCR reactions using equal amounts of cDNA and primers specific for *cysK*, *cysE*, *cysT*, *cysA *and *cysW *were analyzed on a 1% agarose gel (Fig 4). All PCR products were between 730 and 930 bp in length, and each experiment was repeated 3 times. As expected for *E. coli *[35], the *cysE *transcript, which served as an internal control, was expressed equally under all 4 conditions whether L-cysteine was present or absent (Lanes 1-4). The *cysK *transcript was sometimes but not always expressed at higher levels in the absence of L-cysteine (Lanes 1-2) than in the presence of L-cysteine (Lanes 3-4). The transcripts for *cysA *and *cysT *were consistently expressed at higher levels in the absence of L-cysteine (Lanes 1-2) than in its presence (Lanes 3-4). Finally, the transcripts for *cysW *were not always detectable but when they were visible, they were only observed in samples from cells grown in the absence of L-cysteine (Lanes 1-2) and not in samples from cells grown in the presence of L-cysteine (Lanes 3-4). Thus, it appeared that the addition of L-cysteine repressed the expression of the sulfate permease transcripts, *cysA*, *cysT *and *cysW*.

## Discussion

A previous proteomic study on selenite tolerant *E. coli *cells used M-9 minimal medium that lacked L-cysteine [17]. When two derivative K12 strains, MC4100 and GC4468, growing at logarithmic phase were pretreated with 0.25 mM selenite for 60 minutes and then exposed to 25 mM selenite, they were able to tolerate the increased selenite concentrations and were even able to grow for one hour. After this point, the cells entered into a stationary phase and began to die. This increased tolerance was attributed to higher expression levels of superoxide dismutase in response to selenite induced oxidative stress. The current work carried these *E. coli *studies one step further by demonstrating that *Enterobacter *sp. YSU was also sensitive to selenite in M-9 minimal medium, but the selenite inhibition was relieved by supplementing the medium with L-cysteine (Figs 1 and 2).

The role of L-cysteine in the observed selenite resistance/sensitivity phenotypes was investigated using proteomics. Interestingly, spot 7 from cells grown under the selenite sensitive conditions (NCS) contained peptides for CysK (Fig 3 and Table 1), whereas the same spot from cells grown under selenite resistant conditions (CS) did not. This result supported the hypothesis that L-cysteine conferred selenite resistance to *Enterobacter *sp. YSU by preventing selenite uptake through the non-specific pathway or sulfate permease system. The presence of L-cysteine probably caused feedback inhibition of CysE, which converted serine to O-acetylserine [30,35]. Then, lower levels of the O-acetylserine, the derivative of N-acetylserine, which acted as an inducer for the L-cysteine synthesis genes, resulted in decreased expression of *cysK *and the sulfate transport genes. Although the RT-PCR experiments did not conclusively show that the addition of L-cysteine to the growth medium reduced the level of *cysK *transcripts, they did suggest that L-cysteine indirectly repressed the expression of the sulfate transport genes. By decreasing the level of uptake through the sulfate permease system, L-cysteine allowed the cells to grow even when the selenite concentrations were 40 times higher than the sulfate concentrations.

Under the selenite sensitive conditions (NCS), *Enterobacter *sp. YSU appeared to respond to selenite by expressing a putative tellurium resistance protein C [5,34] in spot 8. Since tellurium is located directly under selenium in the periodic table of elements and is chemically similar to selenium [36], this protein may have been expressed non-specifically in response to selenite but was not successful in conferring resistance. The association of a GroES-like protein with the tellurium resistance protein and the appearance of the small heat shock protein in unique spot 9 suggested that these cells were probably experiencing selenite-induced oxidative stress [17].

The function of other identified proteins in spots 7, 10 and 11, which were present at higher intensities in cultures grown in the presence of selenite (NCS and CS) than in cultures grown in the absence of selenite (NCNS and CNS), was not clear. They all contained peptides for 3 proteins: outer membrane protein II (OmpA) from *E. aerogenes *[37], OmpA from *E. sakazakii *and a putative component membrane hydrogenase of OmpA. Basic Local Alignment Search Tool (BLAST) analysis and the references associated with the BLAST results did not provide any additional information about these polypeptides. However, an anaerobic strain of *Clostridium pasteurianum *reduced selenite using a hydrogenase during anaerobic respiration [38], and other Gram negative bacteria such as *Stenotrophomonas maltophilia *and *Enterobacter *sp. SLD1a-1 reduced selenite and selenate and deposited elemental selenium just inside or outside the cell surface [21,39]. Since the three proteins in spots 7, 10 and 11 shared many of the same peptides (Table 1), they may form a single OmpA-like protein that reduced selenite to elemental selenium using the hydrogenase component. Further studies are needed to understand the role that they played in selenite resistance.

## Conclusion

Viable cell count and turbidometric growth curves in M-9 minimal medium showed that *Enterobacter *sp. YSU required L-cysteine to be resistant to 40 mM selenite. Selenite can enter *E. coli *through a specific, undefined pathway and a non-specific sulfate permease pathway [30]. Proteomic and RT-PCR analysis of *Enterobacter *sp. YSU cultures grown in the absence of L-cysteine and presence of selenite (NCS) and in the presence of L-cysteine and selenite (CS) suggested that L-cysteine conferred selenite resistance by feedback inhibition of the synthesis of N-acetylserine. This intermediate in L-cysteine synthesis acted as an inducer for *cysK *and the sulfate permease genes, *cysA*, *cysT*, and *cysW*. The lower levels of inducer decreased the expression of sulfate permease and may have limited selenite transport into the cells through the non-specific pathway, allowing the bacteria survive. This work linked studies on selenite tolerance in M-9 medium lacking L-cysteine [17] with research on selenite transport in *E. coli *[30].

## Methods

### Bacterial strain and media

M-9 minimal medium [40] was described previously, and 5X M-9 Salts were obtained from Becton, Dickinson and Company (Sparks, MD). When required, M-9 medium was supplemented with 0.04 mg/ml L-cysteine (Fisher Scientific, Fair Lawn, NJ) and 40 mM sodium selenite (MP Biomedicals, Aurora, OH). Luria-Bertani (LB) medium [40] and agar were obtained from Fisher Scientific. *Enterobacter *sp. YSU was described previously [32].

### Viable cell count growth curves

Four *Enterobacter *sp. YSU cultures, two containing L-cysteine and two lacking L-cysteine, were grown in M-9 minimal overnight at 37°C and diluted 1:20 into corresponding fresh M-9 minimal medium containing or lacking L-cysteine. These four new cultures were grown at 37°C with shaking. After 1.5 hours of growth, selenite or an equal volume of water was added to give the following culture conditions: no L-cysteine and no selenite (NCNS), no L-cysteine and selenite (NCS), L-cysteine and no selenite (CNS), and L-cysteine and selenite (CS). Samples from each culture were removed every 45 minutes, serially diluted and plated on LB-agar medium in triplicate. Plates were incubated overnight at 37°C, and colony forming units (CFUs) were counted.

### Proteomic analysis growth curves

Two *Enterobacter *sp. YSU cultures, one containing L-cysteine and the other lacking L-cysteine, were grown in M-9 minimal overnight at 37°C and diluted 1:20 into corresponding fresh M-9 minimal medium containing or lacking L-cysteine. These new cultures were grown at 37°C with shaking, and turbidity was measured every 0.5 hour using a Klett Colorimeter with a KS-54 filter. After 2.5 hours of growth, the two cultures were divided into equal volumes. Sodium selenite or an equal volume of water was added to give the four NCNS, NCS, CNS and SC growth conditions. Immediately before and one hour after the addition of sodium selenite, samples were harvested by centrifugation at 5,000 × g and 4°C for 10 minutes, and cell pellets were stored at -80°C.

### Protein extraction

Cells were thawed and resuspended in lysing buffer containing 8 M urea, 2 M thiourea, 2% (w/v) 3-[(3-cholamidopropyl)dimethy-ammonio]-1-propanesulfonate (CHAPS), 2% (w/v) SB 3-10, 40 mM tris(hydroxymethyl)aminomethane (Tris) (Amresco, Solon, OH), 0.2% (v/v) Bio-Lyte^® ^3/10 ampholyte (Bio Rad), 1% (v/v) tributylphosphine (TBP) (Bio Rad) and 1% (v/v) Halt Protease Inhibitor Cocktail (Pierce, Rockford, IL). Cells were lysed using a MiniBeadbeater-8 (BioSpec, Bartlesville, OK) and mixed to final concentrations of 0.2 μg/ml RNase (Amresco) and 200 U/ml DNase I (Pierce). After centrifuging the lysate for 10 minutes at 16,000 × g, the supernatant was treated with Bio-Rad's 2D Clean Up Kit™ (Bio-Rad) and the final pellet was resuspended in rehydration buffer containing 8 M urea, 1% (w/v) CHAPS, 15 mM dithiothreitol (DTT), trace Bromophenol Blue (Amresco), and 0.2% (w/v) Bio-Lyte^® ^3/10 ampholyte (Amresco). A modified Bradford assay [41,42] was used to determine protein concentrations before 2DGE analysis.

### Two-dimensional gel electrophoresis (2DGE)

Isoelectric focusing [43] was carried out using a Bio-Rad Protean IEF Cell (Bio-Rad). A total of 150 μg of protein was separated using an 11 cm IPG Ready Strip (Bio-Rad) with a fixed pH range of 4-7. After active rehydration at 50 V and 20°C for 12 hours, the sample was focused, starting at 0 V and ending at 8,000 V for a total of 40,000 volt hours.

After focusing, the IPG Ready Strip was washed with Equilibration Buffer I containing 6 M urea, 2% (w/v) SDS, 0.375 M Tris-HCl pH 8.8, 20% (v/v) glycerol and 130 mM DTT for 10 minutes and with Equilibration Buffer II containing 6 M urea, 2% (w/v) SDS, 0.375 M Tris-HCl pH 8.8, 20% (v/v) glycerol, and 135 mM iodoacetamide for an additional 10 minutes. The IPG strip was then submerged in 1× tris glycine SDS (TGS) buffer containing 0.025 M tris base, 0.192 M glycine and 0.1% (w/v) sodium dodecyl sulfate (SDS) (Amresco) before being placed in a 10.5%-14% Criterion precast gel (Bio-Rad) for protein size separation at 100 V.

### Staining, imaging, spot selection and protein identification

After size separation, gels were stained with SYPRO^® ^Ruby Protein Stain (Bio-Rad), imaged using the Bio-Rad Gel Chemidoc™ XRS Gel Documentation System and analyzed for matchsets using the Bio-Rad PD Quest 2-D Image Analysis Software [42]. The matchsets were used to select and excise protein spots which were sent to The Ohio State University Proteomics Facility to be digested with trypsin and analyzed by capillary-liquid chromatography-nanospray tandem mass spectrometry (Nano-LC/MS/MS). The sequences of the peptide fragments were analyzed by the Mascot software package (Matrix Science Inc., Boston, MA) to determine the potential identity of the proteins in each excised spot [32].

### RT-PCR

Cells were grown and harvested as in Fig 2. RNA was extracted using Bio-Rad's Aurum™ Total RNA Mini Kit, and cDNA from 0.5 μg of purified RNA was synthesized using Bio-Rad's iScript™ Select cDNA Synthesis Kit. PCR reactions containing 1.5 μl of cDNA, GoTaq^® ^Green Master Mix (Promega, Madison, WI) and primers for *cysK *(5'-CTCGCTGACTATCGGTCA-3' and 5'-GATACCCGCAAGAATACC-3'), *cysA *(5'-ATGAGCATTGAGATTGCC-3' and 5'-ACGACTAATTGGGTGTAG-3'), *cysT *(5'-GCTGTTTGTGTGCCTGAT-3' and 5'-CGACTTTGCAGAGTGTTA-3'), *cysW *(5'-CGTGCAGGCGTTCAGCAA-3' and 5'-CCTGTTGCGCGCGTTTTT-3') or *cysE *(5'-ATGCCGTGTGAAGAACTG-3' and 5'-CTCGAAGGTATGGTGAAT-3') were performed for 30 cycles of 95°C for 1 minute, 50°C for 1 minute and 72°C for 1 minute. Equal volumes of the resulting PCR reactions were analyzed using a 1% agarose (Amresco) gel.

## Competing interests

The authors declare that they have no competing interests.

## Authors' contributions

AJ performed the turbidometric growth curves and proteomic studies. SK, NS, SM and VK worked with NB to perform the viable cell count growth curves. JC performed the RT-PCR experiments. These studies were conceived by JC. The manuscript was written by JC using sections of theses by AJ and NB. All authors read and approved the final manuscript.

## References

[B1] Burk RF (1991). Molecular biology of selenium with implications for its metabolism. FASEB J.

[B2] Foster CB (2004). Selenoproteins and the metabolic features of the archaeal ancestor of eukaryotes. Mol Biol Evol.

[B3] Heider J, Bock A (1993). Selenium metabolism in micro-organisms. Adv Microb Physiol.

[B4] Stadtman TC (1996). Selenocysteine. Annu Rev Biochem.

[B5] Zannoni D, Borsetti F, Harrison JJ, Turner RJ (2008). The bacterial response to the chalcogen metalloids Se and Te. Adv Microb Physiol.

[B6] Forstrom JW, Zakowski JJ, Tappel AL (1978). Identification of the catalytic site of rat liver glutathione peroxidase as selenocysteine. Biochemistry (NY).

[B7] Zinoni F, Birkmann A, Stadtman TC, Bock A (1986). Nucleotide sequence and expression of the selenocysteine-containing polypeptide of formate dehydrogenase (formate-hydrogen-lyase-linked) from *Escherichia coli*. Proc Natl Acad Sci USA.

[B8] Turner RJ, Weiner JH, Taylor DE (1998). Selenium metabolism in *Escherichia coli*. Biometals.

[B9] Takahata M, Tamura T, Abe K, Mihara H, Kurokawa S, Yamamoto Y, Nakano R, Esaki N, Inagaki K (2008). Selenite assimilation into formate dehydrogenase H depends on thioredoxin reductase in *Escherichia coli*. J Biochem.

[B10] Kim IY, Veres Z, Stadtman TC (1992). *Escherichia coli *mutant SELD enzymes. The cysteine 17 residue is essential for selenophosphate formation from ATP and selenide. J Biol Chem.

[B11] Leinfelder W, Forchhammer K, Veprek B, Zehelein E, Bock A (1990). *In vitro *synthesis of selenocysteinyl-tRNA(UCA) from seryl-tRNA(UCA): involvement and characterization of the *selD *gene product. Proc Natl Acad Sci USA.

[B12] Forchhammer K, Bock A (1991). Selenocysteine synthase from *Escherichia coli*. Analysis of the reaction sequence. J Biol Chem.

[B13] Leinfelder W, Zehelein E, Mandrand-Berthelot MA, Bock A (1988). Gene for a novel tRNA species that accepts L-serine and cotranslationally inserts selenocysteine. Nature.

[B14] Li C, Reches M, Engelberg-Kulka H (2000). The bulged nucleotide in the *Escherichia coli *minimal selenocysteine insertion sequence participates in interaction with SelB: a genetic approach. J Bacteriol.

[B15] Sandman KE, Tardiff DF, Neely LA, Noren CJ (2003). Revised *Escherichia coli *selenocysteine insertion requirements determined by *in vivo *screening of combinatorial libraries of SECIS variants. Nucleic Acids Res.

[B16] Terada A, Yoshida M, Seko Y, Kobayashi T, Yoshida K, Nakada M, Nakada K, Echizen H, Ogata H, Rikihisa T (1999). Active oxygen species generation and cellular damage by additives of parenteral preparations: selenium and sulfhydryl compounds. Nutrition.

[B17] Bebien M, Lagniel G, Garin J, Touati D, Vermeglio A, Labarre J (2002). Involvement of superoxide dismutases in the response of *Escherichia coli *to selenium oxides. J Bacteriol.

[B18] Seko Y, Imura N (1997). Active oxygen generation as a possible mechanism of selenium toxicity. Biomed Environ Sci.

[B19] Spallholz JE, Hoffman DJ (2002). Selenium toxicity: cause and effects in aquatic birds. Aquat Toxicol.

[B20] Shamberger RJ (1985). The genotoxicity of selenium. Mutat Res.

[B21] Dungan RS, Yates SR, Frankenberger WT (2003). Transformations of selenate and selenite by *Stenotrophomonas maltophilia *isolated from a seleniferous agricultural drainage pond sediment. Environ Microbiol.

[B22] Mergeay M, Nies D, Schlegel HG, Gerits J, Charles P, Van Gijsegem F (1985). *Alcaligenes eutrophus *CH34 is a facultative chemolithotroph with plasmid-bound resistance to heavy metals. J Bacteriol.

[B23] Roux M, Sarret G, Pignot-Paintrand I, Fontecave M, Coves J (2001). Mobilization of selenite by *Ralstonia metallidurans *CH34. Appl Environ Microbiol.

[B24] Sarret G, Avoscan L, Carriere M, Collins R, Geoffroy N, Carrot F, Coves J, Gouget B (2005). Chemical forms of selenium in the metal-resistant bacterium *Ralstonia metallidurans *CH34 exposed to selenite and selenate. Appl Environ Microbiol.

[B25] Ledgham F, Quest B, Vallaeys T, Mergeay M, Coves J (2005). A probable link between the DedA protein and resistance to selenite. Res Microbiol.

[B26] Bebien M, Kirsch J, Mejean V, Vermeglio A (2002). Involvement of a putative molybdenum enzyme in the reduction of selenate by *Escherichia coli*. Microbiology.

[B27] Ranjard L, Nazaret S, Cournoyer B (2003). Freshwater bacteria can methylate selenium through the thiopurine methyltransferase pathway. Appl Environ Microbiol.

[B28] Ranjard L, Prigent-Combaret C, Nazaret S, Cournoyer B (2002). Methylation of inorganic and organic selenium by the bacterial thiopurine methyltransferase. J Bacteriol.

[B29] Lindblow-Kull C, Kull FJ, Shrift A (1985). Single transporter for sulfate, selenate, and selenite in *Escherichia coli *K-12. J Bacteriol.

[B30] Muller S, Heider J, Bock A (1997). The path of unspecific incorporation of selenium in *Escherichia coli*. Arch Microbiol.

[B31] Lacourciere GM, Levine RL, Stadtman TC (2002). Direct detection of potential selenium delivery proteins by using an *Escherichia coli *strain unable to incorporate selenium from selenite into proteins. Proc Natl Acad Sci USA.

[B32] Holmes A, Vinayak A, Benton C, Esbenshade A, Heinselman C, Frankland D, Kulkarni S, Kurtanich A, Caguiat J (2009). Comparison of two multimetal resistant bacterial strains: *Enterobacter *sp. YSU and *Stenotrophomonas maltophilia *ORO2. Curr Microbiol.

[B33] Perkins DN, Pappin DJ, Creasy DM, Cottrell JS (1999). Probability-based protein identification by searching sequence databases using mass spectrometry data. Electrophoresis.

[B34] Tarr PI, Bilge SS, Vary JC, Jelacic S, Habeeb RL, Ward TR, Baylor MR, Besser TE (2000). Iha: a novel *Escherichia coli *O157:H7 adherence-conferring molecule encoded on a recently acquired chromosomal island of conserved structure. Infect Immun.

[B35] Kredich NM (1996). Biosynthesis of cysteine. Escherichia coli and Salmonella Cellular and Molecular Biology.

[B36] Emsley J (1995). The Elements.

[B37] Chen R, Schmidmayr W, Kramer C, Chen-Schmeisser U, Henning U (1980). Primary structure of major outer membrane protein II (OmpA protein) of *Escherichia coli *K-12. Proc Natl Acad Sci USA.

[B38] Yanke LJ, Bryant RD, Laishley EJ (1995). Hydrogenase I of *Clostridium pasteurianum *functions as a novel selenite reductase. Anaerobe.

[B39] Dungan RS, Frankenberger WT (1998). Reduction of selenite to elemental selenium by *Enterobacter cloacae *SLD1a-1. J Environ Qual.

[B40] Ausubel F, Brent R, Kingston RE, Moore DD, Seidman JG, Smith JA, Struhl K (1997). Short Protocols in Molecular Biology.

[B41] Bradford MM (1976). A rapid and sensitive method for the quantitation of microgram quantities of protein utilizing the principle of protein-dye binding. Anal Biochem.

[B42] Chandler JM, Treece ER, Trenary HR, Brenneman JL, Flickner TJ, Frommelt JL, Oo ZM, Patterson MM, Rundle WT, Valle OV, Kim TD, Walker GR, Cooper CR (2008). Protein profiling of the dimorphic, pathogenic fungus, *Penicillium marneffei*. Proteome Sci.

[B43] O'Farrell PH (1975). High resolution two-dimensional electrophoresis of proteins. J Biol Chem.

